# The ESG-FLW Index: A Multidimensional Composite Index for Comparing Food Loss and Waste Valorisation Pathways

**DOI:** 10.3390/foods15152635

**Published:** 2026-07-27

**Authors:** Riccardo Censi, Domizia Vescovo, Riccardo Mazzucchelli, Marco Ruggeri, Roberto Ruggieri, Donatella Restuccia

**Affiliations:** 1 Department of Management, Sapienza University of Rome, Via del Castro Laurenziano 9, 00161 Rome, Italy; riccardo.censi@uniroma1.it (R.C.); domizia.vescovo@uniroma1.it (D.V.); riccardo.mazzucchelli@uniroma1.it (R.M.); donatella.restuccia@uniroma1.it (D.R.); 2 Department of Wellbeing, Health and Environmental Sustainability, Sapienza University of Rome, Via delle Fontanelle, 02100 Rieti, Italy

**Keywords:** food loss and waste, composite indicator, ESG, food redistribution

## Abstract

Food loss and waste (FLW) constitutes a structural challenge for agri-food systems. However, current analytical tools for assessing management and valorisation options remain fragmented across disciplines. Existing frameworks rarely capture within a single structure the environmental, social, and economic–institutional feasibility dimensions needed to compare alternative recovery pathways. This study introduces the ESG-FLW Index, a multidimensional composite index designed to support comparative evaluation of FLW valorisation strategies, with particular attention to recovery for human consumption. The index draws on a comparative analysis of eight established frameworks. It integrates Life Cycle Assessment for the environmental pillar; Techno-Economic Assessment together with compliance, data-quality, scalability, and replicability indicators for the governance pillar; and Social Return on Investment for the social pillar. These components are aggregated through Multi-Criteria Decision Analysis, following methodological guidance for robust composite indicators. The framework aligns explicitly with the Food Waste Hierarchy and SDG Target 12.3. It also includes hurdle criteria to limit the risk that high aggregate scores obscure critical shortcomings. As a proof of concept, the index was applied to the CiboAmico redistribution programme. Redistribution scored substantially higher than composting (85.1 versus 30.7). The ranking remained stable under ±20% weight variations and Monte Carlo simulations (*n* = 1000). The ESG-FLW Index offers a transparent and replicable decision-support tool. It is best suited to relative comparison among options and to making sustainability trade-offs explicit.

## 1. Introduction

**Figure 1:**
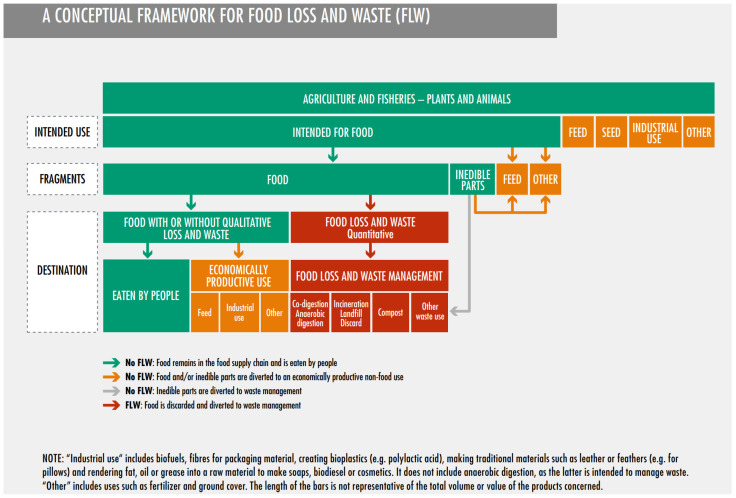
A conceptual framework for food loss and waste (FLW) [9].

Food loss and waste (FLW) constitutes a structural challenge for agri-food systems. It also leaves considerable scope for improvement, with implications for food security and the use of natural resources. The Food and Agriculture Organization of the United Nations (FAO) estimates that approximately one third of food produced for human consumption is lost or wasted along the supply chain. This loss involves inefficient use of natural resources, greenhouse gas emissions, pressure on food security, and reduced economic and social value [1,2]. Wasted food is estimated to account for 8–10% of global greenhouse gas emissions and to occupy 28% of the world’s agricultural land. In addition, food lost because of inadequate cold-chain capacity would be sufficient to feed approximately 1 billion people [3]. In 2015, the United Nations General Assembly included FLW reduction in the Sustainable Development Goals (SDGs). SDG 12.3 aims to halve food waste at retail and consumer levels by 2030. In response, many actors mobilised globally to promote collective action towards this objective [4]. Examples include the Champions 12.3 initiative, which brings together leaders from governments, businesses, and international organisations around a systematic approach to measuring and reducing FLW [5]. This approach has encouraged similar initiatives at regional and national levels. It has also influenced European policies on the circular economy and sustainable food systems, as well as programmes such as the Food Recovery Challenge promoted by the EPA and USDA in the United States [4,6,7,8]. At the conceptual level, the FAO defines FLW as “a decrease in quantity or quality along the food supply chain” [9]. FLW therefore includes losses and waste generated through production, processing, distribution, and consumption (Figure 1). It also includes residual streams that may re-enter circular systems. However, the literature does not provide a single, universally accepted definition of food loss and food waste. As Chaboud and Daviron (2017) suggest, definitions vary according to the problems that actors seek to address [10]. This distinction is therefore not merely taxonomic. It implies different intervention levers and requires evaluative tools that can compare alternative FLW management and valorisation options in multidimensional terms.

**Figure 2:**
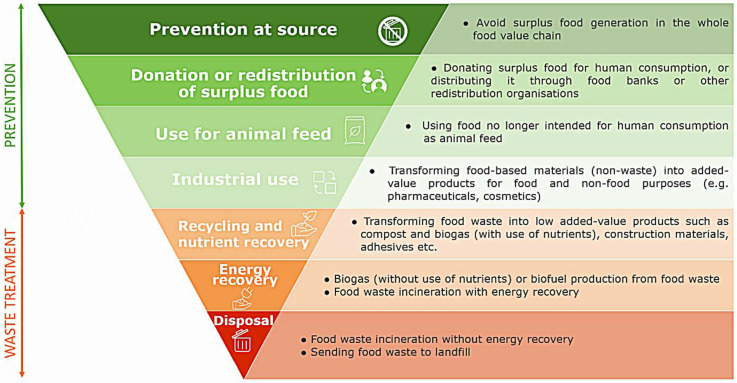
Hierarchy for the prioritisation of options to manage food, by-products from food processing and food waste [11].

The Food Waste Hierarchy is a widely used reference in both policy and research on FLW management (Figure 2) [11]. The framework orders intervention options according to their capacity to maximise the valuable use of food and minimise final disposal. It prioritises prevention at source, followed by donation or redistribution, use as animal feed, industrial use, recycling and nutrient recovery, energy recovery, and disposal as a last resort [11,12]. The U.S. EPA Food Recovery Hierarchy follows a comparable logic and confirms broad international convergence around this hierarchical approach [13].

At the regulatory level, the Food Waste Hierarchy informs several regional and national frameworks. In the European Union, its basis lies in the Waste Framework Directive (2008/98/EC), which establishes a binding order of priority for waste management in all Member States [14]. Two complementary instruments further develop this regulatory architecture. Commission Delegated Decision (EU) 2019/1597 defines a common methodology for measuring FLW in Member States [15]. The EU Platform on Food Losses and Food Waste coordinates prevention measures and monitors progress towards SDG 12.3 [16].

At the international technical and methodological level, the Food Loss and Waste Accounting and Reporting Standard (FLW Standard) provides the global reference methodology for accounting and standardised reporting of loss and waste flows along the supply chain. The FLW Protocol, a multi-stakeholder partnership involving the WRI, FAO, WRAP, and UNEP, developed this standard [17,18]. Despite broad agreement on the importance of reducing FLW, available analytical tools remain fragmented across disciplines. This limitation is widely recognised at both European [18] and international levels [19].

A review of the scientific literature and policy documents makes it possible to identify eight main frameworks, which fall into three analytical traditions:

(1) Quantification and reporting tools, including the FLW Standard and EU Methodology 2019/1597, oriented towards harmonised measurement and institutional reporting of flows [15,17];
(2) Impact and benefit assessment tools, including Life Cycle Assessment (LCA) for the environmental dimension [20], Techno-Economic Assessment (TEA) for economic and institutional feasibility [21,22], Social Return on Investment (SROI) for social benefits [23] and Material Flow Cost Accounting/Material Flow Analysis (MFCA/MFA) for process efficiency and material cost allocation [24,25];
(3) Integrated decision-support tools, including Multi-Criteria Decision Analysis (MCDA) [26,27] and, as an orienting framework, the Food Waste Hierarchy itself [12].

Each tradition has its own object and outputs. However, none provides systematic coverage of all complementary dimensions [18,19,28]. As a result, localised improvements at one stage may be offset by impact shifts along other dimensions of the value chain [2]. This fragmentation reflects the intrinsically interdisciplinary nature of FLW. Beyond technical and logistical determinants, FLW also arises from institutional, organisational, and behavioural factors [29]. Evaluation frameworks therefore need to assess environmental impacts, social benefits, and economic–institutional feasibility at the same time. They should also make trade-offs among objectives and implementation conditions explicit.

ESG (Environmental, Social, Governance) provides an increasingly used structure for multidimensional sustainability assessment. It integrates three core sustainability dimensions within a single conceptual architecture [30,31] in line with emerging non-financial applications of ESG frameworks in public policy and system evaluation. In these applications, the emphasis falls on transparency, multidimensionality, and decision support rather than competitive benchmarking [32,33,34]. This choice reflects the complementarity of the main existing evaluation tools. LCA covers the environmental pillar in a robust manner [32]; SROI captures social benefits associated with redistribution, including equivalent meals and community spillovers [35]; and TEA informs the economic-feasibility component of the governance pillar, while compliance, data quality, scalability, and replicability indicators capture complementary economic–institutional feasibility aspects [30,36]. Among the selected models, no single approach provides a fully operational, transparent, and reusable composite index designed specifically to compare FLW recovery pathways across these three ESG pillars at the same time [18,19].

To translate the ESG framework into a tool that can compare alternatives, composite indices play a central role, as they aggregate heterogeneous indicators into a synthetic measure and facilitate comparison and monitoring in the presence of multiple objectives [37]. In decision-making contexts, the value of the index lies above all in its orienting function (ranking and comparability), which is particularly relevant when evaluating alternative FLW management scenarios [38]. The methodological literature nonetheless identifies several critical issues: indicator selection, normalisation, weighting, and the form of aggregation introduce assumptions that may influence results and create an illusion of precision if they are not adequately stated and tested [37]. For this reason, the OECD/JRC guidelines recommend transparency, traceability of methodological choices, and sensitivity analysis as essential components of a robust composite index [39].

Against this background, this study introduces the ESG-FLW Index. The index is a multidimensional composite indicator designed to support comparative evaluation of FLW valorisation pathways, with particular attention to strategies oriented towards recovery for human consumption. The framework integrates LCA, TEA, and SROI within the environmental, governance, and social pillars, respectively. Within the governance pillar, TEA informs the economic-feasibility component, while compliance, data quality, scalability, and replicability indicators capture broader economic–institutional feasibility. It then aggregates them through Multi-Criteria Decision Analysis (MCDA) into a transparent and replicable ranking system. This approach follows the OECD/JRC guidelines, and aligns explicitly with the Food Waste Hierarchy and SDG Target 12.3. The ESG-FLW Index is primarily intended for decision-makers involved in the management, redistribution, and valorisation of food loss and waste streams. Potential users include public authorities responsible for waste prevention and circular economy policies, food banks and charitable organisations engaged in surplus food redistribution, retailers and food manufacturers evaluating alternative valorisation strategies, and waste management operators involved in the treatment and recovery of organic residues. In addition, the ESG-FLW Index is not intended to replace specialised analytical tools. Rather, it functions as an integrative decision-support instrument that translates heterogeneous sustainability information into an operational and comparable ranking of alternative interventions.

On this basis, this study pursues three specific objectives:

(i) To comparatively analyse eight major frameworks selected for their relevance to FLW quantification, impact assessment, and decision support, identifying complementarities and structural gaps with respect to a multidimensional evaluation of surplus food valorisation pathways;
(ii) To develop the integrated ESG-FLW Index framework following the OECD/JRC methodological guidelines for robust composite indicator construction;
(iii) To test its operational applicability and robustness through a case study based on data from the CiboAmico food redistribution programme (Last Minute Market and Gruppo Hera, Italy) as a methodological proof of concept aimed at verifying the framework’s internal coherence, the traceability of calculation steps, and the index’s operational replicability.

Beyond providing a practical decision-support tool, this study contributes to the methodological literature on FLW assessment by proposing an integrated framework capable of explicitly incorporating environmental and social dimensions together with economic–institutional feasibility within the governance pillar within a transparent and operational ranking system for surplus food valorisation pathways.

## 2. Materials and Methods

This study uses a comparative and integrative analysis of existing frameworks for measuring and managing FLW. The methodological objective is twofold. First, the study assesses the extent to which available tools support multidimensional evaluation of FLW-reduction approaches, including the redistribution of food surpluses that may otherwise become waste. Second, it uses this evidence to develop an integrated decision-support structure.

### 2.1. Comparative Analysis of Existing Models

The study identified relevant models through a targeted, non-systematic review of the scientific literature and policy documents. The analysis covered peer-reviewed articles, grey literature, and normative documents published up to 2025, retrieved from Scopus and Web of Science using the following keywords and Boolean combinations: “food loss and waste assessment”, “FLW framework”, “food surplus valorisation”, “sustainability evaluation food waste”, “ESG food systems”, and combinations thereof. This approach is consistent with scoping review methods used in comparable framework-development studies in the FLW domain [40], where breadth of coverage is prioritised over exhaustive retrieval. Scopus and Web of Science were selected because they are the two most comprehensive multidisciplinary databases for the peer-reviewed literature in the sustainability and environmental-management domain, and their coverage of journals and publishers is complementary rather than fully overlapping; combining them reduces the risk of omitting relevant frameworks indexed in only one database [41]. Given the non-systematic scoping nature of the review, the authors did not apply a formal PRISMA-type deduplication protocol; they compiled records from both databases into a single working list and counted duplicates only once when they were identified during compilation [42]. In the first phase, the targeted review identified models that explicitly address the quantification, evaluation, or valorisation of food surpluses [43,44].

Prior comparative reviews have highlighted the fragmentation of available tools: quantification-focused frameworks such as the WRI FLW Standard and the EU Delegated Decision 2019/1597 operate alongside environmental assessment models (LCA), economic tools (TEA, LCC), and multi-criteria approaches (MCDA, SROI), with few studies attempting an integrated comparison across all three sustainability dimensions [45]. This fragmentation informed the design of the present comparative exercise. The study selected eight representative models on the basis of theoretical relevance, diffusion in the literature, and operational applicability. These models belong to different analytical traditions: the FLW Standard [17], EU Methodology 2019/1597 [15], the Food Waste Hierarchy [12], Life Cycle Assessment [20], Techno-Economic Assessment [21,22], Multi-Criteria Decision Analysis [26,27], Material Flow Cost Accounting/Material Flow Analysis [24,25], and Social Return on Investment [23].

Supplementary Table S1 documents the excluded models and provides an explicit justification for each exclusion. A total of 17 candidate models and approaches were identified through this targeted review; of these, eight met the inclusion criteria and were retained for the comparative analysis, while nine were excluded. The study included models that provided a quantifiable, ESG-relevant methodological contribution applicable to the evaluation of food surplus valorisation or reuse pathways at project or programme level, and that did not duplicate an already included model [46]. The study excluded models that addressed a single dimension already covered by an included model, operated at a macro scale not applicable to individual programmes, were not designed for ESG-type evaluation of FLW scenarios, or could not be implemented as a quantitative tool in the present case. The complete list of candidate models, their inclusion/exclusion status, and the specific justification for each are reported in Supplementary Table S1.

The study systematically analysed each model against eight evaluative constructs. These constructs were derived inductively from recurring thematic categories in the reviewed literature and from policy documents on sustainability assessment in food systems, FLW management and reduction, circular economy approaches applied to the agri-food supply chain, and multi-criteria decision-support frameworks:

1. Quantification and characterisation of losses and waste [47];
2. Alignment with the hierarchy of destinations [48];
3. Environmental sustainability [49];
4. Economic sustainability [49];
5. Social sustainability [49];
6. Governance and policy [50];
7. Technical and organisational feasibility [51];
8. Cultural and behavioural aspects [29].

Table 1 summarises the derivation of these constructs and links each construct to key references in the literature.

The study assessed construct coverage using a four-level qualitative coding scheme: high, medium, low, and none. Comparative framework analyses commonly use this type of scheme to identify complementarities and structural gaps among conceptual models [64]. Two authors independently coded each model–construct pair. They resolved discrepancies through discussion until they reached consensus, following standard inter-rater reliability procedures for qualitative content analysis [65]. Cohen’s kappa quantified agreement between coders on the initial, pre-discussion coding of the 8 × 8 model–construct matrix. The result was κ = 0.81 (95% CI: 0.71–0.91), which corresponds to “almost perfect” agreement under conventional benchmarks for qualitative coding reliability [66]. Disagreements occurred in 12 of the 64 model–construct cells (18.8%). The authors identified them through a systematic cell-by-cell comparison of the two independent coding sheets. They then discussed each discrepant cell with direct reference to the original source material. The assigned code was revised only when both coders agreed on the appropriate qualitative category. The reported coding therefore reflects full consensus rather than a simple majority or averaging rule.

### 2.2. ESG Framing and Methodological Integration

In the second phase, the study mapped the selected models onto the three ESG pillars to assess their relative contribution and identify an integrated conceptual framework. The use of ESG as the reference structure responds to the need to move beyond sectoral approaches and to align the analysis with emerging practices in sustainability assessment and reporting [32]. Within this scheme:

▪ The environmental dimension was linked to LCA, widely used to compare the environmental impacts of prevention, redistribution, and recovery options [54].
▪ The governance pillar was addressed through a set of economic–institutional feasibility indicators. In this study, TEA informs the economic-feasibility component by evaluating costs and financial viability, while scalability and replicability are captured through dedicated governance indicators [52,57].
▪ The social dimension was represented through SROI. This approach is increasingly applied to food redistribution programmes to quantify social benefits in terms of equivalent meals, community value, and inclusion [67]. SROI was selected because it translates heterogeneous social outcomes into a comparable metric, enabling integration within a composite index [35]. Alternative approaches were considered, but they present limitations in terms of comparability, data availability, and operationalisation. Although SROI implies normative assumptions linked to the monetisation of social value, its use is consistent with the nature of the proposed framework [35].

The study integrated heterogeneous metrics through MCDA, a tool widely used in sustainability assessments and waste management. MCDA allows the aggregation of different types of indicators while making explicit the normative assumptions underlying weighting choices [68,69].

The construction of the ESG-FLW Index follows the OECD guidelines and the methodological recommendations of the European Commission’s Joint Research Centre. These references support transparency, reproducibility, and robustness through sensitivity analysis [39]. The framework has an explicitly evaluative and normative nature. It supports public decisions that are consistent with the Food Waste Hierarchy and SDG 12.3, rather than functioning as a neutral descriptive tool.

### 2.3. Construction of the Composite Index and Robustness Analysis

**Figure 3:**
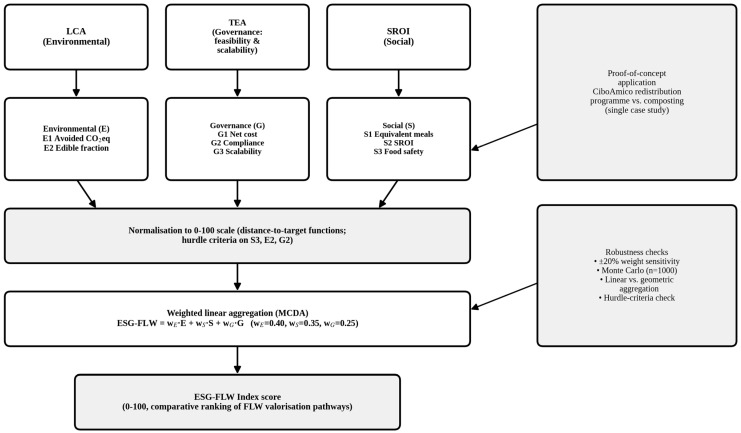
Schematic overview of the ESG-FLW Index framework: the three analytical tools (LCA, TEA, SROI) feed the environmental, governance, and social pillars and their component indicators.

The third phase covered indicator selection, normalisation, weighting, and aggregation (Figure 3). The case-study input data are organised in a structured dataset in the Supplementary Material. The dataset includes both raw values and normalised indicators to support transparency and reproducibility. A minimum set of core indicators was defined:

▪ For the environmental pillar, avoided CO_2_ eq emissions and the share of recovered edible fraction [70];
▪ For the social pillar, equivalent meals distributed, the SROI ratio, and food safety incidents [71];
▪ For the governance pillar, net cost, a compliance and data quality score, and a scalability/replicability index [72].

The study normalised indicators on a 0–100 scale through objective functions based on scientific and policy benchmarks [73]. The study adopted linear normalisation because consolidated evidence on non-linear dynamics is not yet available. Alternative approaches may become relevant in contexts characterised by threshold effects [74]. For an indicator to be maximised, normalisation is expressed as in Equation (1):

(1) $$N_{i}=\min\left(\frac{x_{i}}{T_{i}}\times 100,\;100\right)$$

where:

$N_{i}$ = normalised score for indicator i (on a 0–100 scale);
$x_{i}$ = actual value observed for indicator i;
$T_{i}$ = target value.

For indicators to be minimised, such as costs or food safety incidents, the study adopted an inverse function while retaining the same reference scale. Specifically, indicators to be minimised were normalised as

(2) $$N_{i}\;=\max\;\{[(W_{i}-x_{i})/(\;W_{i}-T_{i})]\;\times \;100,\;0\}$$

where $W_{i}$ denotes a defined worst-case reference value for indicator i, $T_{i}$ the target value, and $x_{i}$ the observed value; results below 0 or above 100 were capped to preserve consistency with the 0–100 scale used for indicators to be maximised. The worst-case reference value for net cost (G1) adopted in the case study is 0.033 €/kg. This value is consistent with the redistribution and composting scores. Target values do not represent absolute scientific thresholds; rather, they function as policy-based comparative references that are consistent with the literature and with the regulatory objectives considered [75].

Pillar weights were set at 0.40 (environmental), 0.35 (social), and 0.25 (governance), reflecting the priorities expressed in European strategies [76,77]. Sensitivity analysis tested alternative configurations, which did not alter the relative ordering of scenarios. These specific numerical weights are not directly prescribed by the cited European strategies. Those strategies express broad thematic priorities rather than quantitative allocations across ESG pillars. The adopted values therefore constitute an author-defined weighting scheme, informed by and consistent with those priorities, rather than values derived from a single authoritative source [78]. Future applications of the framework could strengthen the legitimacy of the weighting scheme through structured expert consultation or participatory multi-stakeholder weighting exercises [39,79].

The study calculated each pillar score as the mean of the normalised values of the indicators assigned to that pillar (Equation (3)).

(3) $$P=\frac{1}{n_{P}}\sum_{i=1}^{n_{P}}N_{i}$$

where:

P = score of the ESG pillar considered (E, S, or G);
$N_{i}$ = normalised score of indicator *i*;
$n_{p}$ = number of indicators included in the pillar considered.

The study then obtained the overall ESG-FLW Index score through weighted linear aggregation of the three pillars, as shown in Equation (4).

(4) $$ESG\text{-}FLW=w_{E}\cdot E+w_{S}\cdot S+w_{G}\cdot G$$

where:

$w_{E}$, $w_{S}$, $w_{G}$ = weights assigned to environmental, social, and governance pillars respectively;
E, S, G = composite scores of each respective pillar.

The study also tested geometric aggregation exclusively for sensitivity-analysis purposes [80]. Hurdle criteria were introduced for food safety, recovered edible fraction, and regulatory compliance to reduce the risk that high aggregate performance might obscure critical shortcomings [81]. Operationally, hurdle criteria were applied as pass/fail checks anchored to the target values reported in Supplementary Table S5: a scenario failing to meet the minimum acceptable performance on any hurdle indicator was flagged as such in the interpretation of results, irrespective of its aggregate score, consistent with the hurdle-criteria logic of preventing compensatory masking in composite indicators [82]. For food safety incidents (S3), any incident above zero constitutes an automatic hurdle failure. For recovered edible fraction (E2), the composting scenario in the case study failed the hurdle by recording a 0% recovery rate, i.e., a complete absence of edible-food recovery for human consumption [83]; this is treated as an automatic failure regardless of the minimum threshold otherwise applied to partial-recovery scenarios.

Finally, the study conducted sensitivity and uncertainty analyses, including variations in pillar weights (±20%) and Monte Carlo simulations (*n* = 1000) on uncertain inputs. The simulations assumed uniform distributions within literature-based ranges because available information was insufficient to estimate empirical distributions.

The study has a predominantly methodological purpose. The case study serves as a proof of concept to test the framework’s internal coherence, operational reproducibility, and robustness [84]. Some indicators, especially social-pillar indicators and governance-pillar economic–institutional feasibility indicators, rely on secondary data or reasoned authorial attributions [85]; the index is therefore better suited to relative comparisons, or ranking, than to absolute score assessment.

The data and parameters used in the analysis are available in the Supplementary Material. They include the complete case-study input dataset (Supplementary Table S2), Monte Carlo simulation parameters (Supplementary Tables S3 and S4), the mathematical formulation of the index (Supplementary Table S5), and the normalisation targets with their sources and rationale (Supplementary Table S6). Figure 3 summarises the overall architecture of the framework from the three analytical tools to the final index score.

## 3. Results

### 3.1. Comparative Analysis of Existing Models

The first analytical phase examined how the selected models address strategies for redistributing food surpluses. Table 2 presents a comparative synthesis of their scope, redistribution orientation, outputs, strengths, and main limitations.

The results indicate an evident distinction between tools mainly oriented towards reporting and tools oriented towards impact assessment. In particular, the FLW Standard and the EU 2019/1597 methodology are effective for measuring, harmonising, and comparing FLW, but they do not provide an evaluative framework for the impacts associated with different destinations for surpluses beyond flow accounting [86]. The Food Waste Hierarchy gives explicit priority to prevention and redistribution. However, it functions mainly as a prescriptive guide and does not produce a quantitative output for comparing options [87].

Among assessment tools, LCA provides established evidence on the comparative environmental impacts of redistribution and alternative options. TEA estimates the economic feasibility of redistribution scenarios in terms of costs and financial sustainability [88]. SROI explicitly captures the social benefits of redistribution pathways, although it does not include environmental or economic dimensions [71]. Finally, MCDA can integrate heterogeneous metrics into a ranking system. This integration remains sensitive to weighting choices and decision assumptions [68].

### 3.2. Gap Analysis Across Constructs

Table 3 summarises each model’s coverage of the eight constructs considered. The results show that no model covers all dimensions needed for a multidimensional evaluation of redistribution. Quantification is well supported by the FLW Standard, the EU Methodology, and MFCA/MFA, whereas environmental assessment is fully ensured only by LCA (and partially by MFCA/MFA) [54]. TEA mainly addresses economic feasibility, while SROI captures social benefits explicitly. MCDA incorporates these benefits only in an integrative form [67]. Governance and policy aspects appear more systematically in compliance and reporting frameworks, such as the FLW Standard and the Food Waste Hierarchy, and in decision procedures such as MCDA. Cultural and behavioural aspects receive only limited and non-systematic attention, with the partial exception of SROI [68].

Overall, the gap analysis indicates a pattern of fragmentation consistent with the literature. Available tools tend to cover individual sustainability dimensions, while the need remains for an integrated evaluation that includes environmental impacts, social benefits, and economic–institutional feasibility at the same time.

### 3.3. Mapping onto the ESG Pillars

Table 4 presents the mapping of the models onto the ESG dimensions.

The results indicate that LCA covers the environmental pillar most directly. TEA is mainly associated with the economic-feasibility component of the governance pillar, while scalability, replicability, compliance, and data quality are treated as complementary economic–institutional feasibility indicators. SROI is centred on the social pillar. TEA should therefore be understood primarily as an economic-feasibility tool [21,22,23], and its contribution to the governance pillar remains narrower than the full scope of governance commonly used in ESG frameworks [89], which also encompasses transparency, accountability, stakeholder participation, and institutional control. Within the ESG-FLW Index, TEA informs the economic-feasibility component of the governance pillar, while regulatory compliance, data quality, scalability, and replicability are captured through dedicated governance indicators. Together, these indicators operationalise the economic–institutional feasibility construct used in this study. Broader governance dimensions, such as stakeholder participation and institutional accountability, are not currently represented in the index. This limitation should be addressed in future refinements. The FLW Standard, EU 2019/1597, and the Food Waste Hierarchy contribute mainly to governance through regulatory alignment and reporting. MCDA can integrate all three dimensions if assumptions about weights, aggregation functions, and decision criteria are made explicit. These findings support the ESG-FLW Index as an instrument for integrating complementary analytical tools rather than replacing individual approaches.

## 4. Case Study: Application of the ESG-FLW Index (Proof of Concept)

The study tested the operational applicability of the proposed framework through a case study based on real data from the CiboAmico initiative, promoted by Last Minute Market and Gruppo Hera in Italy. The programme redistributes food surpluses from corporate canteens to charitable associations. It may therefore contribute to waste prevention and generate environmental and social benefits. According to the 2025 report, the initiative recovered 280.8 tonnes of edible products in one year, generating about 17,000 equivalent meals and avoiding 561,600 kg of CO_2_ eq emissions [90]. The study selected CiboAmico on the basis of three explicit criteria. First, publicly documented operational data cover the environmental and social dimensions together with economic–institutional feasibility within the governance pillar, a combination that is rarely available for food redistribution programmes. Second, the initiative has an established multi-year operational history, which provides a realistic dataset rather than evidence from a short-term pilot. Third, composting offers a directly comparable alternative destination for the same food surplus, allowing the index’s discriminant capacity to be tested under contrasting conditions. Programmes without simultaneous coverage of the three ESG dimensions, or without a directly comparable alternative scenario, were therefore not considered suitable for this proof-of-concept application. Consistent with the methodological purpose of the study, a single data-rich case study was preferred to a broader but shallower multi-case comparison. This choice prioritised traceability and reproducibility of the calculation chain over statistical generalisability. Future research should extend the empirical base to multiple redistribution programmes across different European contexts. Where possible, this should include programmes already assessed through alternative frameworks, in order to support direct comparative benchmarking.

The case study serves as a proof of concept for methodological rather than statistical purposes [91,92]. Its objective is to verify the framework’s internal coherence, the traceability of transformations, and the operational replicability of the calculations. The data were organised in a structured dataset in the Supplementary Material. This dataset includes input values, normalised indicators, and aggregated scores.

### 4.1. Input Data and Derivation of Indicators

Supplementary Table S2 reports the complete case-study input dataset, including raw values for both the redistribution and composting scenarios. On the basis of the available information, the study calculated the following indicators per kilogram of recovered food:

▪ Avoided CO_2_ eq (E1): 561,600 kg CO_2_ eq/280,800 kg = 2.0 kg CO_2_ eq/kg [1];
▪ Recovered edible fraction (E2): conservatively set at 95% [1,53];
▪ Equivalent meals (S1): 16,790/280,800 ≈ 0.060 meals/kg (authors’ calculation based on the FAO (2013)) [1];
▪ SROI (S2): assumed to be 3.38 [53];
▪ Food safety incidents (S3): 0 [90];
▪ Net cost (G1): assumed to be −0.05 €/kg on the basis of sectoral evidence [53];
▪ Compliance and data quality (G2): 80/100;
▪ Scalability/replicability (G3): 85/100.

Some values, particularly compliance and scalability, derive from authorial estimates and secondary data rather than direct measurement. More specifically, expert elicitation informed the compliance and data quality score (G2) and the scalability/replicability index (G3). These indicators function as proxies because standardised, empirically validated metrics for these governance-pillar economic–institutional feasibility indicators are not available at the case-study level. The G2 score of 80/100 reflects the authors’ qualitative assessment of the programme’s partial compliance with the EU 2019/1597 measurement requirements, benchmarked against full compliance (100/100). The G3 score of 85/100 is a conservative estimate based on the operational and organisational characteristics of the CiboAmico programme. It is benchmarked against a theoretical reference for fully replicable programmes and cross-checked against comparable evidence on food-waste-management scalability reported in the literature [52,57]. Because these scores derive from qualitative expert judgement rather than a point-by-point numerical rubric, they should be interpreted as informed approximations rather than precise measurements. The study states these assumptions explicitly for transparency and treats them as sources of uncertainty in the sensitivity analysis and Monte Carlo simulations. Future applications could progressively replace these proxy scores with primary data, such as third-party compliance audits for G2 or documented replication cases and structured organisational-capacity assessments for G3. This would strengthen the empirical grounding of the governance pillar. Composting was considered as the comparison scenario [1], assuming 0.2 kg CO_2_ eq avoided/kg, no recovered edible fraction and no equivalent meals, and an average cost of +0.02 €/kg.

### 4.2. Indicator Normalisation

The study normalised indicators on a 0–100 scale through target-based functions [93]. For avoided emissions, a target of 1.0 kg CO_2_ eq/kg was adopted; redistribution (2.0 kg/kg) therefore reached saturation (100). For equivalent meals, a target of 1 meal/kg was set, producing a score of 6 for redistribution. The Monte Carlo iterations applied the same normalisation functions consistently. Table 5 reports the normalised scores.

The target values adopted for the case study reflect the available literature and policy references rather than universally fixed thresholds. Supplementary Table S6 documents these values in full and reports, for each indicator, the target source, benchmark type, and brief rationale. Briefly, the 1.0 kg CO_2_ eq/kg target for avoided emissions (E1) is a conservative threshold derived from average avoided-emission factors for redistribution versus disposal reported in the FAO’s global assessment [1]. The target for recovered edible fraction (E2) is set at 100%, as an ideal best-practice reference that avoids artificial score saturation at intermediate values. The target for equivalent meals (S1), 1.0 meals/kg, corresponds to the FAO conversion factor for redistributed mixed-product baskets. The SROI target (S2) of 3.0 reflects a sector benchmark for efficient redistribution programmes rather than a universal threshold. The target for food safety incidents (S3) is zero, reflecting a mandatory regulatory requirement under which any incident lowers the score. The net cost target (G1), ≤0 €/kg, represents the break-even point relative to the alternative disposal cost and acts as a threshold for economic self-sufficiency. The targets for regulatory compliance (G2) and scalability/replicability (G3), both set at 100/100, represent theoretical references for full regulatory compliance and fully replicable programmes, respectively. The case-study scores of 80 and 85 were benchmarked against these references as authors’ estimates. Consistent with the distance-to-target normalisation logic adopted throughout the framework [75], these values should be understood as transparent and adjustable calibration references rather than absolute scientific thresholds. Users applying the ESG-FLW Index to other contexts should recalibrate targets against locally relevant benchmarks.

### 4.3. ESG-FLW Index Scores and Interpretation

The study calculated pillar scores as the simple arithmetic mean of the normalised indicators belonging to each pillar, as defined in Equation (3); the pillar weights reported in Section 2.3 were applied only at the subsequent aggregation stage, in Equation (4). The results are as follows:

▪ Scenario A (redistribution): E = 97.5; S ≈ 68.7; G ≈ 88.3; overall index = 85.1;
▪ Scenario B (composting): E = 10; S = 33.3; G = 60; overall index = 30.7.

The ESG-FLW Index produces a marked separation between the two scenarios analysed. Consistent with the normative orientation of the framework, which gives priority to prevention and reuse [12], the redistribution scenario shows high scores in the environmental and social pillars and exceeds the minimum thresholds for edibility, safety, and compliance. The composting scenario receives a lower score and fails to meet the threshold for recovered edible fraction. It is therefore classified as “non-compliant” under the defined hurdle criteria. This outcome largely reflects the design of the framework. The selected indicators, pillar weights, and hurdle criteria are explicitly constructed to prioritise recovery for human consumption, in line with the Food Waste Hierarchy [12]. Accordingly, the comparison should not be read as independent empirical evidence of the universal superiority of redistribution over composting. Rather, it demonstrates that the index reproduces its normative premises within a real application context.

These results should be interpreted as outputs conditioned by the assumptions of the framework, rather than as absolute or universally generalisable evaluations of the options considered [39,73]. More specifically, the case study verifies the operational coherence of the index and its capacity to make priorities and trade-offs transparent in a real application context [84,92].

### 4.4. Sensitivity Analysis and Ranking Robustness

The sensitivity analysis assessed the stability of the case-study results in relation to the main methodological assumptions and input uncertainty. The analysis considered three dimensions:

(i) ESG pillar weights;
(ii) Variability of quantitative indicators, including avoided CO_2_ eq, costs, and SROI;
(iii) Aggregation form.

Varying pillar weights by ±20% relative to baseline values, sampled independently and renormalised to sum to unity, produced index values between 82.8 and 87.2 for redistribution and between 27.7 and 33.8 for composting. In all cases, the two scenarios remained clearly separated. Monte Carlo simulations (*n* = 1000) were conducted on the uncertain quantitative inputs. These included avoided CO_2_ eq and net cost for both scenarios, and SROI for redistribution. The study sampled these inputs independently from uniform distributions bounded by the literature-based minimum and maximum values reported in Supplementary Table S3. The simulations also included independent, renormalised variation in the three pillar weights within the same ±20% range. The remaining indicators—recovered edible fraction, equivalent meals, food safety incidents, regulatory compliance, and scalability/replicability—were held at their baseline values, as reported in Supplementary Table S2, because no literature-based uncertainty ranges were available for them. The simulations returned a mean score of 84.8 (SD ±1.1; 95% range 82.3–86.6) for redistribution and 30.2 (SD ±3.3; 95% range 25.0–36.7) for composting. No iteration reversed the ranking between the two scenarios. Finally, geometric aggregation, tested for sensitivity purposes, confirmed the stability of the distinction between scenarios. This result suggests that the outcome does not critically depend on the aggregation form adopted.

Overall, within the scope of the present case study, the results suggest that the index has sufficient internal robustness to function as an orienting tool for relative comparison, or ranking, among options. The analysis also makes explicit the implications of normative choices and the sensitivity of results to weights and uncertainty. Broader claims of reliability or generalisability would require validation on additional cases.

## 5. Discussion

### 5.1. Methodological Implications of the ESG-FLW Index

The comparative analysis indicates that the available frameworks are highly specialised when considered individually, but remain incomplete when assessed together [94]. This finding has primarily methodological relevance. By integrating LCA, TEA, and SROI through MCDA, the framework shifts the evaluation from separate domain-specific assessments to a structured comparative procedure. In this process, outputs expressed in non-commensurable units become comparable through normalisation, weighting, and aggregation. Each step involves explicit methodological choices and value-based assumptions [37,39]. Consequently, the informational value of the ESG-FLW Index lies in its orienting function, namely the production of a transparent and traceable ranking, rather than in the absolute quantification of performance [38]. Previous studies have generally applied these tools separately, for example by using LCA to compare food-waste treatment options [55,56] or SROI to assess specific redistribution programmes [58,71].

More specifically, LCA does not provide social evidence or direct evidence for the governance pillar, SROI does not provide environmental evidence or direct evidence for the governance pillar, and TEA does not provide environmental or social evidence. TEA instead informs the economic-feasibility component of the governance pillar, while compliance, data quality, scalability, and replicability indicators capture broader economic–institutional feasibility. None of these tools can therefore produce, on its own, a single ranking across redistribution and disposal pathways. The ESG-FLW Index addresses this gap by combining the three complementary evidence bases into one traceable score. Its contribution lies in the joint operationalisation of specialised assessment tools within an integrative decision-support architecture.

This integrative structure also raises a methodological issue. Weighted linear aggregation is inherently compensatory, meaning that strong performance in one pillar may offset a substantial weakness in another. As a result, an apparently acceptable aggregate score may coexist with poor performance on a specific dimension [82]. The ESG-FLW Index addresses this risk through hurdle criteria. Food safety, recovered edible fraction, and regulatory compliance are assessed through pass/fail conditions that operate independently of the aggregate score. These criteria reduce the risk that favourable environmental or economic results compensate for failure to meet requirements treated as non-negotiable [81,83].

The proof-of-concept application illustrates this mechanism. Although the composting scenario showed a marginally more favourable regulatory-compliance profile, it was classified as non-compliant because it recovered no edible fraction for human consumption. A purely compensatory aggregation could have absorbed this limitation within the final score. By limiting such compensation, the ESG-FLW Index differs from standard additive composite indices and aligns the overall assessment with the prescriptive logic of the Food Waste Hierarchy.

However, hurdle criteria constrain compensation only across the dimensions to which they are applied. Because each pillar score is calculated as the mean of its normalised indicators, compensation remains possible within pillars and among indicators not subject to hurdle requirements. The safeguard is therefore selective rather than general. The scope of the hurdle set therefore becomes a substantive design choice. Extending or narrowing it changes the balance between compensatory flexibility and non-compensatory rigour. This trade-off should be made explicit whenever the framework is reconfigured for a different evaluation context.

### 5.2. Contextual Calibration and Transferability of the ESG-FLW Index

The scores produced by the index depend on the target values used to normalise each indicator. Because the framework adopts a distance-to-target approach, the same observed performance may receive different normalised scores when assessed against different benchmarks. Benchmark selection is therefore not merely technical; it directly shapes the results [73,75]. The ESG-assessment literature recognises this issue, since differences in methodological design can lead to divergent ratings for the same entity [95,96]. In the present case study, the targets are policy-based comparative references derived from the literature and regulatory objectives, rather than an absolute scientific threshold. This has implications for interpretation. The magnitude of a score can only be understood in relation to the benchmark against which it is calculated. This confirms that the index is better suited to relative comparison among alternatives than to absolute performance assessment. In addition, linear normalisation assumes that indicators do not exhibit threshold or saturation effects. Where such effects are likely, non-linear normalisation functions supported by empirical evidence may provide a more appropriate representation [74].

These considerations also define the conditions under which the index may be extended beyond the present case. The proof-of-concept application addressed a single FLW stream. It did not test alternative FLW streams, such as manufacturing or household waste, or alternative valorisation pathways, such as animal feed or industrial reuse. These options follow different regulatory, logistical, and value-recovery logics and may therefore yield different pillar-level trade-offs [6]. A direct empirical comparison with other European organisations that have applied alternative FLW assessment frameworks would be valuable for benchmarking the contribution of the ESG-FLW Index. However, it falls outside the scope of the present proof-of-concept study, which is limited to demonstrating internal coherence and operational replicability on a single data-rich case. Similarly, the stability of the index under alternative aggregation functions, linear and geometric, supports its internal robustness within the present application. Its reliability and applicability as a comparative tool across a broader range of redistribution pathways, however, remain to be established through additional validation [73,80].

The pillar weights reflect a normative orientation consistent with European food-waste priorities. When the index is applied in contexts shaped by different policy objectives, regulatory settings, or stakeholder expectations, these weights should be recalibrated through context-specific elicitation procedures. Structured expert consultation or participatory multi-stakeholder approaches would be preferable [68,69,76]. The use of secondary and literature-based data for several social-pillar indicators and governance-pillar economic–institutional feasibility indicators also limit the extent to which scores can be interpreted in absolute terms. Primary data would be needed to support a more robust assessment of performance beyond relative comparison. Cultural and behavioural determinants also require further attention, since they remain among the least systematically represented dimensions in both the reviewed frameworks and the ESG-FLW Index. Their integration becomes particularly important when redistribution outcomes depend on consumer acceptance, organisational practices, or behavioural change [29]. Under these conditions, the index can provide public authorities, redistribution organisations, and food operators with a transparent and replicable basis for comparing valorisation options, in line with the Food Waste Hierarchy [12,60] and SDG Target 12.3.

Beyond these operational and methodological conditions, the ESG-FLW Index also has a broader societal dimension. By making explicit the trade-offs between environmental-pillar outcomes, social-pillar outcomes, and economic–institutional feasibility within the governance pillar, the framework can support decisions that affect food security, access to food for vulnerable populations, community resilience, and the distribution of social value generated by surplus-food recovery. In this sense, the index’s contribution may extend beyond individual organisations or supply chains. By providing a transparent basis for prioritising redistribution over lower-value destinations such as composting, it can inform public funding and procurement decisions that scale up food-recovery efforts. Its societal relevance is particularly evident in relation to food-poverty alleviation and the Sustainable Development Goals.

### 5.3. Practical Implications, Adoption Conditions, and Contextual Applicability

Beyond its methodological contribution, the ESG-FLW Index may have different practical implications across geographical and institutional contexts. In developed countries, where regulatory reporting systems, redistribution networks, and ESG disclosure practices are often more established, the framework can support public procurement, funding prioritisation, corporate sustainability reporting, and the comparison of alternative food-waste valorisation pathways. In developing countries, its use may be more closely linked to food-security objectives, infrastructure constraints, informal redistribution channels, and the need to prioritise low-cost interventions with high social value. In both contexts, the framework should not be applied as a fixed universal scoring system, but as an adaptable decision-support tool whose targets, weights, and indicators are recalibrated according to local data availability, regulatory conditions, food-chain structure, and stakeholder priorities.

Different actors may benefit from the framework in different ways. Governments can use it to support policy design, SDG 12.3 monitoring, and funding decisions. NGOs and food banks can use it to demonstrate the social, environmental, and operational value of redistribution activities. Companies, retailers, restaurants, and food-service operators can use it to compare prevention, redistribution, recycling, and disposal options and to identify improvement priorities. Consumers and society may benefit indirectly through increased transparency, reduced environmental impacts, improved food access, and greater awareness of food-waste consequences. However, implementation entails costs, including data collection, staff time, training, and possible expert support for calibration and weighting. These costs may be reduced through simplified templates, integration with existing reporting systems, and phased implementation.

The adoption of the ESG-FLW Index by supply-chain actors cannot be assumed automatically. Acceptance will depend on perceived usefulness, administrative burden, data availability, trust in the scoring procedure, regulatory incentives, and the extent to which actors are involved in adapting the framework to their context. For restaurants and smaller establishments, a simplified version would be necessary, focusing on a limited number of easily measurable indicators such as edible surplus, avoided disposal, redistribution partners, food-safety compliance, and basic cost data. Uptake could be accelerated through pilot applications, user-friendly guidance, digital calculation templates, training activities, and participatory calibration involving governments, companies, NGOs, food-service operators, and consumer organisations.

## 6. Conclusions

Food loss and waste remains a multidimensional challenge involving environmental-pillar outcomes, social-pillar outcomes, and economic–institutional feasibility within the governance pillar. The comparative analysis showed that available analytical tools tend to address these dimensions separately. Reporting-oriented frameworks, such as the FLW Standard and the EU Methodology, are effective for quantification and monitoring purposes, while LCA, TEA, and SROI provide detailed assessments of specific sustainability dimensions. However, no single framework offers a transparent and operational mechanism for evaluating and comparing alternative FLW valorisation pathways across these dimensions at the same time.

To address this gap, this study developed the ESG-FLW Index. The index is a multidimensional composite indicator that integrates environmental and social pillar outcomes with economic–institutional feasibility indicators within the governance pillar. By combining LCA, TEA, and SROI through a structured MCDA approach, the framework supports operational comparison among alternative FLW management strategies. It also makes normative assumptions, priorities, and sustainability trade-offs explicit. The proof-of-concept application to the CiboAmico food redistribution programme indicated the practical feasibility of the approach. The resulting ranking remained stable under sensitivity and uncertainty analyses.

The main scientific contribution of this work lies in the development of a composite indicator and in showing why single-dimension approaches are insufficient for assessing FLW valorisation pathways. Environmental performance, social value creation, and economic–institutional feasibility within the governance pillar emerges as complementary and interdependent dimensions. Their interactions should therefore be evaluated together. In this respect, the ESG-FLW Index contributes to the methodological literature on FLW assessment by providing a transparent, replicable, and policy-oriented framework for comparative sustainability evaluation.

From a practical perspective, and within the scope demonstrated by the present proof-of-concept application, the ESG-FLW Index can support evidence-based decision-making. It helps identify options that are consistent with the Food Waste Hierarchy and SDG Target 12.3 by translating heterogeneous sustainability information into a comparable ranking system. For public authorities, the index may offer a replicable basis for procurement and funding-prioritisation decisions, consistent with accountability requirements in European ESG reporting regulations [49]. For food operators and redistribution organisations, the inclusion of scalability and replicability as governance-pillar economic–institutional feasibility indicators can support performance self-assessment and inform decisions on programme expansion [72].

Several limitations should nonetheless inform the interpretation of these results. First, the framework was tested on a single case study. Although the *CiboAmico* programme is representative of the Italian food-redistribution context, the proof-of-concept design demonstrates operational applicability and internal coherence rather than statistical validation or generalisability. The resulting scores and ranking therefore cannot be automatically transferred to other supply chains, product categories, or geographical settings. Future research should apply the index to multiple FLW streams, such as retail, manufacturing, and household waste, and to alternative valorisation pathways, including animal feed, industrial reuse, anaerobic digestion, energy recovery, and disposal. Such applications would help test the framework’s external robustness and transferability.

Second, data quality and representativeness constrain the precision of the estimates, particularly for the environmental component. The LCA parameters for avoided emissions derive largely from secondary sources and literature benchmarks. These sources are standard in comparative studies, but they may not capture local specificities. Several social-pillar indicators and governance-pillar economic–institutional feasibility indicators were also estimated from literature averages or authorial attributions because complete primary data were not available. Given the weight of the social dimension, this epistemic uncertainty makes the index more reliable for relative comparison than for absolute assessment of individual performances. Sensitivity and Monte Carlo analyses mitigate this uncertainty, but they do not eliminate it.

Third, the weighting of the ESG pillars reflects a normative approach aligned with European food-waste priorities rather than a universally valid scheme. Although the ranking remained stable under substantial weight variations, participatory weighting could strengthen the framework’s legitimacy in real decision-making contexts.

Finally, the analysis is predominantly static. It relies on aggregated annual data and does not capture the temporal dynamics that may shape medium-to-long-term trade-offs. Integrating dynamic or multi-period models therefore represents a promising development. Relatedly, the index is intentionally oriented towards prevention and redistribution, in line with the Food Waste Hierarchy and SDG Target 12.3. This orientation is normative rather than neutral. It privileges options with high social value and lower environmental impact, which may limit its applicability as a general analytical tool where such priorities are not shared. Public decision-makers and analysts should therefore interpret the results in light of the framework’s policy-oriented function. They should use the index to support, rather than replace, in-depth method-specific evaluations.

## Figures and Tables

**Table 1:** Evaluative constructs used.

Construct	Description	Source
Quantification and characterisation	Measurement of FLW quantities and types	[15,18,52]
Alignment with the hierarchy	Consistency with the priorities of the Food Waste Hierarchy	[12,53]
Environmental sustainability	Assessment of environmental impacts (e.g., GHG emissions, resource use)	[54,55]
Economic sustainability	Cost–benefit analysis, profitability, and economic efficiency	[56,57]
Social sustainability	Impacts on communities, inclusion, and access to food	[58]
Governance and policy	Presence of regulatory instruments, compliance, and accountability	[59]
Technical and organisational feasibility	Implementation capacity, replicability, and scalability	[52,60]
Cultural and behavioural aspects	Attitudes, behaviours, and cultural barriers	[61,62,63]

**Table 2:** Comparative overview of FLW models relevant to redistribution pathways.

Model/Standard	Scope	Orientation Towards Redistribution	Output	Strengths	Limitations
FLW Standard	FLW quantification and reporting	Indirect (enables destination tracking)	Flow balances	Comparable and policy-aligned	No impact assessment
EU 2019/1597 [15]	Harmonised EU monitoring	Indirect (includes preparation for reuse)	Supply-chain-stage KPIs	Regulatory compliance and harmonisation	Limited detail on the benefits of redistribution
Food Waste Hierarchy	Policy and priority framework	Direct (redistribution)	Qualitative ranking	Simple and intuitive	No quantitative impact assessment
LCA (ISO 14040/44)	Environmental impact assessment	Direct (scenario-based)	CF, WF, LU, energy	Rigorous and comparative	Data-intensive and allocation-sensitive
TEA	Economic feasibility	Direct (scenario-based)	Costs, payback, NPV	Economic clarity	No environmental or social dimension
MCDA	Decision-support tool	Direct (integrates scenarios)	Weighted rankings	Integrative and flexible	Sensitive to subjective weights
MFCA/MFA	Process efficiency and costs	Indirect (identifies hotspots)	Flow/cost allocation	Highlights losses	Limited system-level perspective
SROI	Social impact assessment	Direct (redistribution-focused)	Social € per € invested; meals	Captures social value	Difficult to standardise; lacks E/G dimensions

**Table 3:** Gap matrix of models against the evaluation constructs for redistribution.

Construct	FLW Standard	EU 2019/1597	Food Waste Hierarchy	LCA	TEA	MCDA	MFCA/MFA	SROI
Quantification	High	High	Low	Medium	Medium	Medium	High	Medium
Alignment with waste hierarchy	Medium	Medium	High	Medium	Medium	High	Medium	Medium
Environmental dimension	Low	Low	Low	High	Medium	High	Medium	Low
Economic dimension	Low	Low	None	Medium	High	High	Medium	Low
Social dimension	Low	Low	Low	Medium	Low	High	Low	High
Governance/policy	High	High	High	Medium	Medium	High	Medium	Medium
Technical feasibility	Medium	Medium	Low	Medium	High	High	High	Medium
Cultural/behavioural	None	None	None	None	None	Medium	None	High

**Table 4:** Alignment of models with the ESG pillars.

Model	Environmental (E)	Social (S)	Governance (G)	Overall ESG Coverage
FLW Standard	High	Low	High	Medium
EU 2019/1597	High	Low	High	Medium
Food Waste Hierarchy	Medium	Medium	High	Medium
LCA	High	Medium	Low	High (E-focused)
TEA	Medium	Low	High	High (G-focused)
SROI	Low	High	Medium	High (S-focused)
MFCA/MFA	High	Low	Medium	Medium
MCDA	High	High	High	Potentially high

**Table 5:** Normalised indicator scores for redistribution vs. composting.

Indicator	Redistribution (Scenario A)	Composting (Scenario B)	Target/Benchmark	Normalised Values (A vs. B)
E1—Avoided CO_2_ eq (kg/kg)	2.0	0.2	1.0	100 vs. 20
E2—Edible fraction (%)	95	0	100	95 vs. 0
S1—Equivalent meals (per kg)	0.060	0	1.0	6 vs. 0
S2—SROI	3.38	0	3.0	100 vs. 0
S3—Food safety incidents	0	0	0 (optimal)	100 vs. 100
G1—Net cost (€/kg)	−0.05	+0.02	≤0	100 vs. 40
G2—Compliance (0–100)	80	90	100	80 vs. 90
G3—Scalability (0–100)	85	50	100	85 vs. 50

## Data Availability

All data supporting the findings of this study are contained within the article and its Supplementary Materials. The complete case-study input dataset, including both raw values and normalised indicators (Supplementary Table S2), the Monte Carlo simulation parameters (Supplementary Table S3), the mathematical formulation of the index (Supplementary Table S4), and the normalisation targets with their sources and rationale (Supplementary Table S5) are provided as Supplementary Material, together with the documentation of the models excluded from the selection (Supplementary Table S1). No new data were created beyond those reported in the article and in the Supplementary Material. Any further information is available from the corresponding author upon reasonable request.

## Supplementary Materials

The following supporting information can be downloaded at: https://www.mdpi.com/article/10.3390/foods15152635/s1, Table S1: Model selection; Table S2: Case study input dataset; Table S3: Parameters for sensitivity analysis and Monte Carlo simulations; Table S4. Monte Carlo simulation and weight-sensitivity results (*n* = 1000); Table S5: Mathematical formulation of the ESG-FLW Reuse Index; Table S6: Normalization targets: source, type, and rationale [97,98,99,100,101,102,103,104,105,106,107].

## Abbreviations

The following abbreviations are used in this manuscript:

LCA	Life Cycle Assessment
MCDA	Multi-Criteria Decision Analysis
MFCA/MFA	Material Flow Cost Accounting/Material Flow Analysis
OECD	Organisation for Economic Co-operation and Development
SDG	Sustainable Development Goal
SROI	Social Return on Investment
TEA	Techno-Economic Assessment
UNEP	United Nations Environment Programme
WRAP	Waste and Resources Action Programme
WRI	World Resources Institute
