# Supplementary material for: The ESG-FLW Index: A Multidimensional Composite Index for Comparing Food Loss and Waste Valorisation Pathways
Source: Foods. 2026 Jul 27;15(15):2635. doi: 10.3390/foods15152635 (PMC13464748; doi:10.3390/foods15152635)
Supplement: Supplementary file 1 [file foods-15-02635-s001.zip › foods-4395933-supplementary.pdf]

Supplementary Materials

Table S1. Model selection

Model / Approach	Included in the study	Justification for selection / exclusion	Source
Agent-Based Modeling (ABM)	No	A predictive approach useful for dynamic simulations, but not designed for the ESG evaluation of FLW scenarios.	[1]
Analytic Hierarchy Process (AHP)	Yes	Used to calculate weights in MCDA with stakeholder involvement.	
Carbon Footprint (CF)	No	Treated as an LCA output, not as a standalone framework.	[2]
Cost-Benefit Analysis (CBA)	No	Covers only the economic dimension and is therefore insufficient for an integrated ESG analysis.	[3]
Ecological Footprint	No	Macro-scale indicators that are poorly suited to local and operational contexts.	[4]
Food Waste Hierarchy (EU / US EPA)	Yes	A normative model included among the relevant frameworks for reuse.	
Human Development Index (HDI)	No	A macro-social indicator not applicable to individual projects or initiatives.	[5]
Life Cycle Assessment (LCA)	Yes	A core model for comparative environmental assessment; included.	
Life Cycle Costing (LCC)	No	Already absorbed within the logic of Techno-Economic Assessment (TEA).	[6]
Marginal Abatement Cost Curves (MACC)	No	An approach useful for climate analysis, but not applicable to food reuse.	[7]
Material Flow Cost Accounting (MFCA / MFA)	Yes	Included as a tool for analyzing material flows and process costs.	
Multi-Criteria Decision Analysis (MCDA)	Yes	Essential for integrating ESG dimensions; included in the framework.	
Social Return on Investment (SROI)	Yes	The only model focused on social value; included.	

Techno-Economic Assessment (TEA)	Yes	Essential for assessing costs, scalability, and economic feasibility; included.	
Triple Bottom Line (TBL)	No	An inspiring concept, but not implementable as a quantitative tool for this case.	[8]
Water Footprint (WF)	No	Treated as a single environmental indicator already included in LCA.	[9]
WRI FLW Standard	Yes	Reference framework for the report	

Table S2. Case study input dataset

Variable	Unit	Redistribution	Composting	Source
Quantity of food treated	kg/year	280.800	280.800	[10]
CO_2e avoided, total	kg CO_2e	561.600	56.160	[11]
Specific CO_2e avoided	kg CO_2e/kg	2.0	0.2	Authors' calculation based on FAO (2013) [11]
Recovered edible fraction	%	95	0	[11,12]
Total equivalent meals	n	16.790	0	[10]
Specific equivalent meals	meals/kg	0.060	0	Authors' calculation based on FAO (2013) [11]
SROI	ratio	3.38	0	[12]
Food safety incidents	n	0	0	[10]
Net cost	€/kg	-0.05	+0.02	[12]
Regulatory compliance	score (0-100)	80	90	Authors' estimate
Scalability/replicability	score (0-100)	85	50	Authors' estimate

Table S3. **Parameters for sensitivity analysis and Monte Carlo simulations; (b)**

Variable	Scenario	Baseline	Min	Max	Distribution
Specific CO_2e avoided	Redistribution	2.0	1.5	2.5	Uniform
Net cost	Redistribution	-0.05	-0.10	0.00	Uniform
SROI	Redistribution	3.38	2.5	4.5	Uniform
Specific CO_2e avoided	Composting	0.2	0.1	0.3	Uniform
Net cost	Composting	0.02	0.00	0.05	Uniform
w_E	All	0.40	0.32	0.48	Uniform
w_S	All	0.35	0.28	0.42	Uniform
w_G	All	0.25	0.20	0.30	Uniform

Table S4. **Monte Carlo simulation and weight-sensitivity results (n = 1000).**

Analysis	Scenario	Mean	SD	Min	Max	95% range
Weight sensitivity (±20%, renormalised)	Redistribution	85.1	–	82.8	87.2	–
Weight sensitivity (±20%, renormalised)	Composting	30.7	–	27.7	33.8	–
Full Monte Carlo (n = 1000)	Redistribution	84.8	1.1	81.1	87.1	82.3–86.6
Full Monte Carlo (n = 1000)	Composting	30.2	3.3	23.4	40.1	25.0–36.7

Note: results computed from the parameters reported in Table S3, applying Equations 1-3 (main text). Avoided CO_2eq and net cost (both scenarios) and SROI (redistribution) were sampled independently from the uniform distributions in Table S3; pillar weights were sampled independently within ±20% of baseline and renormalised to sum to 1. Recovered edible fraction, equivalent meals, food safety incidents, regulatory compliance, and scalability/replicability were held at their baseline values (Table S2), as no literature-based uncertainty ranges were available for them. Ranking between scenarios did not reverse in any of the 1000 iterations.

Table S5. **Mathematical formulation of the ESG-FLW Reuse Index**

Level	Formula
Pillar E	E = (E1 + E2) / 2
Pillar S	S = (S1 + S2 + S3) / 3
Pillar G	G = (G1 + G2 + G3) / 3
ESG-FLW Index	$ESG = w_E \cdot E + w_S \cdot S + w_G \cdot G$
Geometric aggregation	$ESG_{geo} = E^{wE} \cdot S^{wS} \cdot G^{wG}$

Table S6. Normalization targets: source, type, and rationale

Indicator	Code	Target	Target source	Type	Brief rationale
CO_2e avoided	E1	1.0 kg CO_2e/kg	Authors based on FAO (2013) [11]	Normative-authorial	A conservative threshold derived from average avoided-emission factors for redistribution versus disposal [11]; values >1.0 are capped at 100
Recovered edible fraction	E2	100%	[12]	Best practice	Full recovery is used as the ideal reference; this avoids artificial score saturation at intermediate values
Equivalent meals	S1	1.0 meals/kg	Authors based on FAO (2013) [11]	Normative-authorial	This corresponds to approximately 1 meal per kg of food, a threshold consistent with the FAO conversion for redistributed mixed-product baskets
SROI	S2	3.0	[12]	Sector benchmark	SROI value observed by WRAP for efficient redistribution programs; not a universal threshold, but the most relevant sector benchmark available
Food safety incidents	S3	0	[13]	Mandatory regulations	Zero incidents are a legal requirement; inverse function: 0 incidents = 100, any incident lowers the score
Net cost	G1	≤0 €/kg	[12]	Economic-sectoral	Break-even relative to the alternative disposal cost; threshold for the program's economic self-sufficiency

Regulatory compliance	G2	100/100	Authors based on EU 2019/1597 [14]	Normative	Full compliance with EU FLW measurement requirements; the score of 80 for redistribution reflects the authors' estimate of partial compliance
Scalability/replicability	G3	100/100	Authors based on Caldeira et al. (2019); Garrone et al. (2016) [15,16]	Operational-authorial	Theoretical reference for fully replicable programs; the score of 85 is a conservative estimate by the authors based on the operational characteristics of the CiboAmico program

References

1. Anggraeni, M.C.; Silaban, C.A.; Anggreainy, M.S.; Cahyadi, E. Role of Artificial Intelligence in the Management of Food Waste. In Proceedings of the 2021 2nd International Conference on Artificial Intelligence and Data Sciences (AiDAS), Perak Branch, Malaysia, 8 September 2021; pp. 1–6.
2. Eriksson, M.; Strid, I.; Hansson, P.-A. Carbon Footprint of Food Waste Management Options in the Waste Hierarchy—A Swedish Case Study. *J. Clean. Prod.* **2015**, *93*, 115–125. https://doi.org/10.1016/j.jclepro.2015.01.026.
3. Lam, C.-M.; Yu, I.K.M.; Medel, F.; Tsang, D.C.W.; Hsu, S.-C.; Poon, C.S. Life-Cycle Cost-Benefit Analysis on Sustainable Food Waste Management: The Case of Hong Kong International Airport. *J. Clean. Prod.* **2018**, *187*, 751–762. https://doi.org/10.1016/j.jclepro.2018.03.160.
4. You, S.; Sonne, C.; Park, Y.-K.; Kumar, S.; Lin, K.-Y.A.; Ok, Y.S.; Wang, F. Food Loss and Waste: A Carbon Footprint Too Big to Be Ignored. *Sustain. Environ.* **2022**, *8*, 2115685. https://doi.org/10.1080/27658511.2022.2115685.
5. United Nations Environment Programm. *Food Waste Index Report 2021*; United Nations Environment Programm: Nairobi, Kenya, 2021; ISBN 978-92-807-3868-1.
6. De Menna, F.; Dietershagen, J.; Loubiere, M.; Vittuari, M. Life Cycle Costing of Food Waste: A Review of Methodological Approaches. *Waste Manag.* **2018**, *73*, 1–13. https://doi.org/10.1016/j.wasman.2017.12.032.
7. Eory, V.; Pellerin, S.; Carmona Garcia, G.; Lehtonen, H.; Licite, I.; Mattila, H.; Lund-Sørensen, T.; Muldowney, J.; Popluga, D.; Strandmark, L.; et al. Marginal Abatement Cost Curves for Agricultural Climate Policy: State-of-the Art, Lessons Learnt and Future Potential. *J. Clean. Prod.* **2018**, *182*, 705–716. https://doi.org/10.1016/j.jclepro.2018.01.252.
8. Singh, S.; Srivastava, S.K. Decision Support Framework for Integrating Triple Bottom Line (TBL) Sustainability in Agriculture Supply Chain. *SAMPJ* **2022**, *13*, 387–413. https://doi.org/10.1108/SAMPJ-07-2021-0264.
9. Hoehn, D.; Margallo, M.; Laso, J.; Ruiz-Salmón, I.; Fernández-Ríos, A.; Campos, C.; Vázquez-Rowe, I.; Aldaco, R.; Quinteiro, P. Water Footprint Assessment of Food Loss and Waste Management Strategies in Spanish Regions. *Sustainability* **2021**, *13*, 7538. https://doi.org/10.3390/su13147538.

10. Last Minute Market. Available online: https://drive.google.com/file/d/1Z3hQPOfMN6BKtPt9rGvCxuVpy7TKsAW9/view (accessed on 4 June 2026).
11. FAO. *Food Wastage Footprint: Impacts on Natural Resources: Summary Report.*; FAO: Rome, Italy. 2013; p. 61, ISBN 978-92-5-107752-8.
12. The Waste and Resources Action Programme. *Annual Report and Consolidated Accounts 2024/2025*; The Waste and Resources Action Programme: Banbury, UK, 2025;
13. European Parliament and Council of the European Union *Regulation (EU) No 178/2002 of the European Parliament and of the Council*; European Parliament and Council of the European Union: Brussels, Belgium, 2002; pp. 1–24.
14. European Parliament. *Commission Delegated Decision (EU) 2019/1597 of 3 May 2019 Supplementing Directive 2008/98/EC of the European Parliament and of the Council as Regards a Common Methodology and Minimum Quality Requirements for the Uniform Measurement of Levels of Food Waste*; European Parliament and Council of the European Union: Brussels, Belgium, 2019.
15. Caldeira, C.; De Laurentiis, V.; Corrado, S.; Van Holsteijn, F.; Sala, S. Quantification of Food Waste per Product Group along the Food Supply Chain in the European Union: A Mass Flow Analysis. *Resour. Conserv. Recycl.* **2019**, *149*, 479–488. https://doi.org/10.1016/j.resconrec.2019.06.011.
16. Garrone, P.; Melacini, M.; Perego, A.; Sert, S. Reducing Food Waste in Food Manufacturing Companies. *J. Clean. Prod.* **2016**, *137*, 1076–1085. https://doi.org/10.1016/j.jclepro.2016.07.145.
